# Substituted Hydroxyapatites with Antibacterial Properties

**DOI:** 10.1155/2014/178123

**Published:** 2014-05-11

**Authors:** Joanna Kolmas, Ewa Groszyk, Dagmara Kwiatkowska-Różycka

**Affiliations:** Department of Inorganic and Analytical Chemistry, Faculty of Pharmacy, Medical University of Warsaw, Ul. Banacha 1, 02-097 Warsaw, Poland

## Abstract

Reconstructive surgery is presently struggling with the problem of infections located within implantation biomaterials. Of course, the best antibacterial protection is antibiotic therapy. However, oral antibiotic therapy is sometimes ineffective, while administering an antibiotic at the location of infection is often associated with an unfavourable ratio of dosage efficiency and toxic effect. Thus, the present study aims to find a new factor which may improve antibacterial activity while also presenting low toxicity to the human cells. Such factors are usually implemented along with the implant itself and may be an integral part of it. Many recent studies have focused on inorganic factors, such as metal nanoparticles, salts, and metal oxides. The advantages of inorganic factors include the ease with which they can be combined with ceramic and polymeric biomaterials. The following review focuses on hydroxyapatites substituted with ions with antibacterial properties. It considers materials that have already been applied in regenerative medicine (e.g., hydroxyapatites with silver ions) and those that are only at the preliminary stage of research and which could potentially be used in implantology or dentistry. We present methods for the synthesis of modified apatites and the antibacterial mechanisms of various ions as well as their antibacterial efficiency.

## 1. Introduction

Calcium phosphates—mainly hydroxyapatites—have for many years played a key role in biomaterial engineering due to their high biocompatibility and their bioactivity in human mineralized tissues, especially in bones and dental mineralized tissues, such as enamel, dentine, and cement [1, 2]. Biological apatite, being the main component of the inorganic fraction, is a carbonate hydroxyapatite, depleted in hydroxyl groups and rich in small amounts of various ions, including Mg^2+^, K^+^, Na^+^, Mn^2+^, HPO_4_^2−^, and SiO_4_^4−^ [1–3].

**Figure 1:**
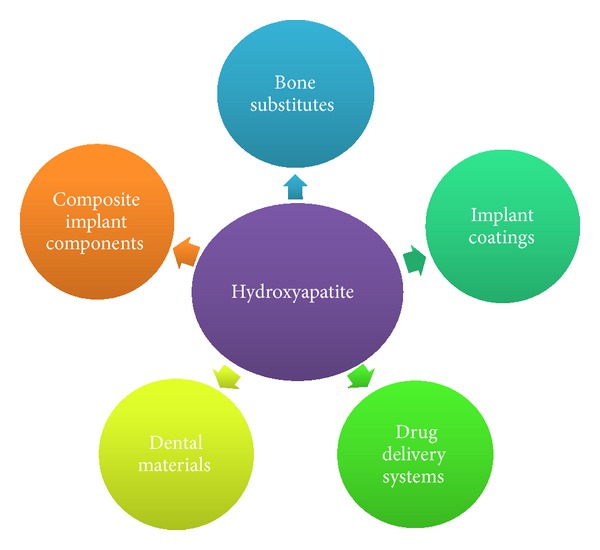
Schematic view of hydroxyapatites' applications.

Synthetic hydroxyapatite (HA, HAp) is used in reconstruction and repair surgery, conservative dentistry, dental implantology, and pharmacy (Figure 1) [1, 4–6].

**Figure 2:**
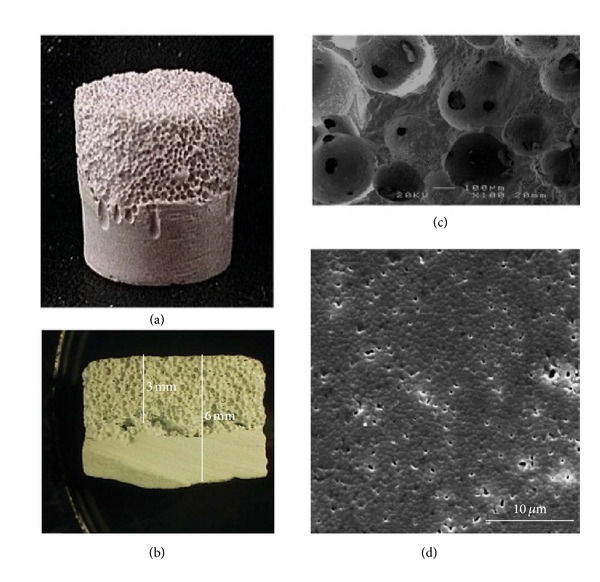
A hemiporous HAp scaffold: (a) overview, (b) cross-section, (c) porous part, and (d) dense part. Reprinted from [7] with permission.

A porous hydroxyapatite material may be used as a substitute bone material that fills tooth sockets after tooth extraction or else as a biomaterial forming a scaffold for newly formed bone (see Figure 2) [7]. Between such material and the bone, the formation of biological fixation takes place: the structure of the living bone tissue penetrates the free space of the material, thus causing the more permanent fixation of the implant in the bone [4].

Due to its poor mechanical properties (brittleness and inflexibility), the use of hydroxyapatite as a substitute bone material is unfortunately limited to places that are not subject to great tension. On the other hand, a hydroxyapatite material is a perfect component of composite implants with synthetic polymers and biopolymers [8–10]. Dense hydroxyapatite bioceramics, formed into suitable shapes, may be used in the creation of implants for the middle ear and eye (orbital implant), as well as PD implants (Percutaneous Device), and are included in inner dialysis systems [11–13]. Hydroxyapatite is widely used as a coating of metallic implants for bones in order to improve and accelerate the process of osseointegration [14, 15]. Hydroxyapatite powder plays an important role in dentistry (e.g., in the treatment of dental pulp and dentine hypersensitivity) and is associated with the exposure of dentinal tubules [16, 17]. Hydroxyapatite, present in toothpastes and dental gels, reduces the deposition of accretions on teeth. It can also be used as a component of dental cements and fillings [18]. It is also worth mentioning that microporous structures of hydroxyapatite can serve as carriers of drugs supplying medicinal substances directly to a destination. The studies on hydroxyapatite systems for the controlled release of anticancer drugs, antibiotics, and growth factors were reported in several papers [19–21].

Hydroxyapatite is a crystalline calcium phosphate of the general formula: Ca_10_(PO_4_)_6_(OH)_2_. Stoichiometric and perfectly pure hydroxyapatite crystallizes in the monoclinic system [22]. Broadly, HAp crystallizes most often in the hexagonal system, a space group of P6/3mc.

**Figure 3:**
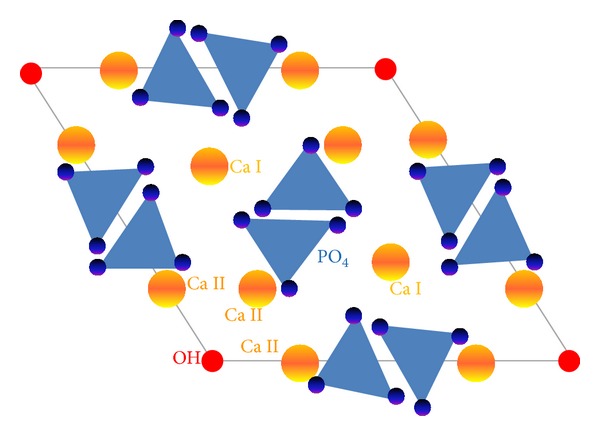
Hydroxyapatite structure—a schematic view (adapted from [1]).

Important structural features of the stoichiometric hydroxyapatite are its structural hydroxyl groups, arranged at the edges of elementary cells, forming the columns –OH–OH–OH—(see Figure 3). Oxygen atoms of these groups are spaced in such a way that they are unable to form hydrogen bonds [23]. Hydroxyapatite includes two types of calcium cations, referred to as Ca (I) and Ca (II). The atoms of calcium Ca (I) are located at the edges of a hexagonal unit cell, while the atoms of calcium Ca (II) form equilateral triangles with the column of structural hydroxyl groups in the middle. The phosphate ions are the largest ions that build unit cells, being the one to determine its structure [24].

One of the primary features of hydroxyapatite is its capacity for ion substitution (i.e., ion exchange). This means that the locations for hydroxyl ions may be occupied by ions of a similar size and charge, such as Cl^−^ or F^−^. In turn, the locations for phosphate ions are occupied by BO_3_^3−^ ions, and the locations for calcium cations by the ions Mg^2+^, Mn^2+^, or Sr^2+^ [1, 25, 26].

In some cases, we are talking about the total exchange (this is possible in the case of the exchange of OH–F or Ca–Sr) or partial exchange (limitations are mostly due to the difference in the sizes of the ions, charges, and their spatial structures).

An important aspect is also the possibility of the substitution of ions with different charges. For example, it is possible to exchange phosphate ions (−3) with carbonate ions (−2). Such a situation leads to the creation of a positively charged vacancy, which is compensated for by the simultaneous release of one cation of calcium (Ca^2+^) and one hydroxyl ion (OH^−^) [27].

This capacity for the ion exchange of hydroxyapatites has been used recently in biomaterial engineering. One also uses the fact that the introduction of even small quantities of some ions may cause changes/improvements in biological, physicochemical, or mechanical properties [28, 29]. For example, the introduction of magnesium ions has a positive biological effect and improves the osseointegrational properties of the material [30]. In turn, the introduction of a small amount of Mn^2+^ ions to the apatite favours osteoblasts proliferation and increases the biocompatibility [31].

It should be emphasized that infections around bone implants comprise the key problem for modern reconstruction surgery [32, 33]. The defence mechanisms of the body must be supported by the introduction of antibacterial factors, particularly antibiotic therapy. This type of therapy is performed using the oral route and is less effective: the dose of the antibiotic must be high in order to ensure its appropriate concentration around the inserted implant. In turn, local antibiotic therapy applied at the place of infection (e.g., at the location of the implant filled with an appropriate antibiotic) also has its limitations: on the one hand, the concentration of the antibacterial drug must be high in order to ensure the efficiency of the treatment; and on the other hand, it must not exceed a safe level of toxicity to cells.

Therefore, implantation surgery seeks additional factors that increase the antibacterial activity around introduced biomaterials.

At present, a fairly common tendency is to use the antibacterial properties of certain ions, including silver (Ag^+^), copper (Cu^2+^), and zinc (Zn^2+^). In addition, one tries to look for the possibility of using other ions, such as cerium Ce^3+^, gallium Ga^3+^, selenium SeO_3_^2−^, titanium Ti^4+^, and strontium Sr^2+^, among others.

**Figure 4:**
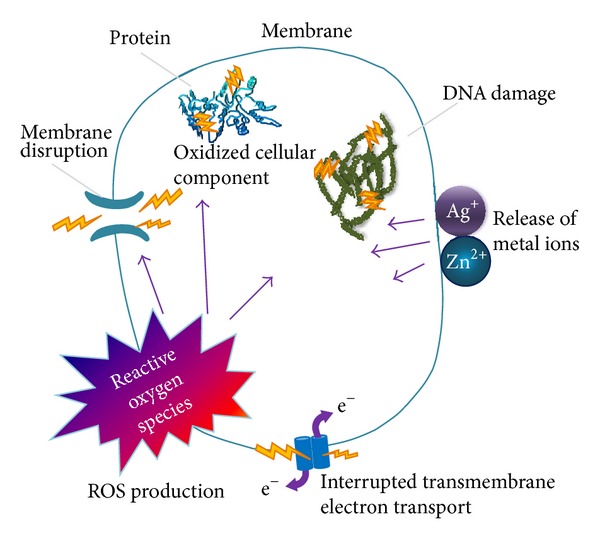
Antibacterial mechanisms of nanoparticles and their ions, adapted from [38] with permission.

The mechanisms of the antibacterial activity of ions are not fully explained. However, the literature provides information on three hypothetical mechanisms [34–37] (Figure 4). According to the first mechanism, the ions penetrate into the bacterial cell and, by affecting the production of intracellular ATP, they disrupt the process of DNA replication. The second mechanism is associated with the accumulation of ions in the cell membranes of bacteria, and thus with changes in their permeability (the gradual release of proteins and lipopolysaccharides). The transportation of protons through the cell membrane is prevented, and consequently it leads to the destruction of the cell membrane and the death of the bacterial cell. The third mechanism is based on the ion induction of reactive oxygen species (ROSs). Oxygen radicals are able to react with the components of the membrane and cell wall of bacteria, as well as other cell components (e.g., mitochondria), causing irreversible changes in their structure and thus the death of the bacterial cell.

The purpose of this paper is to review the available literature and summarize the various achievements in the field of hydroxyapatites modified by ions with antibacterial properties. We have described the achievements concerning the synthesis and testing of the biological and microbiological activity of these compounds. We have tried to present our own point of view as to the applicatory potential of these materials. We hope that our research will serve as a valuable guide to hydroxyapatite bioceramics with additional antibacterial activity.

## 2. Ag^+^-Substituted Hydroxyapatite

For hundreds of years, silver has been widely known for its strong antibacterial properties [39] of an exceptionally broad spectrum, including both gram-positive and gram-negative bacteria, viruses, and fungi [40, 41]. Of all the antibacterial metals, silver has the highest efficiency at concentrations as low as 35 ppb, at which it has no toxic effect on mammalian cells [42, 43]. In addition, microorganisms have a relatively poor ability to develop immunity to silver. Therefore, it is widely used in many forms in order to limit the growth of microorganisms, both in medicine (e.g., for the treatment of flash burns, catheters, the coverage of implant materials, urinary tract infections, dentistry [36, 44] and in other fields [45, 46]).

**Figure 5:**
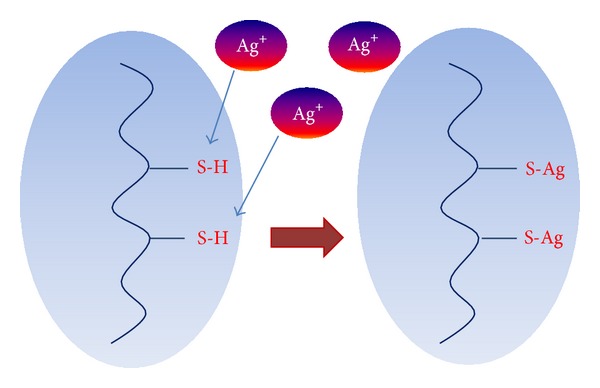
The main proposed mechanism of Ag^+^ ions' antibacterial activity (adapted from [49]).

The mechanism of the action of silver on microbial cells depends upon its form and is complex in each case since it remains insufficiently understood in detail [47]. In the case of silver ions, it is based primarily on their interaction with groups–SH (thiolic) of proteins [48] (see Figure 5), including the exchange of a hydrogen atoms with silver atoms, leading to the formation of S–Ag bonding [49].

This results in the inactivation of both proteins located in the cell membrane and those within the cytoplasm. Following the denaturing action of silver ions, a number of changes occur in the bacteria cell, including dysfunction of the respiratory chain and membrane pumps; the cell membrane shrinks and separates from the cell wall, and as a result the cell contents leak out and the cell wall is torn apart [47, 50]. Silver ions also interact with DNA molecules, causing their condensation and loss of capacity to replicate [47]. It was also shown that silver ions intensify the production of ROSs [51], which undoubtedly enhances their antibacterial properties [49]. In turn, silver in its nanoparticle form acts primarily on the basis of the induction of the formation of ROSs that interact with the cell wall and cell membrane of bacteria, causing their depressurization and an increase in permeability. As a result, the cell content flows out and the cell dies [52–54].

Authors studying the mechanism of the action of Ag^+^ ions suggest that the thickness of the peptidoglycan layer of gram-positive bacteria may—at least to a lesser extent—protect the bacterial cell from the influx of Ag^+^ ions to the inside, which would explain the higher activity of Ag^+^ against G− (e.g.,* Escherichia coli*) as opposed to G+ bacteria (e.g.,* Staphylococcus aureus*) [47, 50].

Increasing attention on the part of researchers has been attracted by hydroxyapatite doped with silver ions (Ag-HAp). It has been shown that Ag-HAp exhibits strong antibacterial activity [52–57], and one has proposed two mechanisms for its activity. The first of them suggests that microorganisms are attracted by electrostatic force to the surface of the hydroxyapatite, where there is a direct interaction between the bacterial cell membrane and silver ions. However, according to the second mechanism, silver ions are slowly released from the interior of the hydroxyapatite, which discloses its bactericidal activity throughout the material surrounding it [55].

There are various methods for the preparation of Ag-HAp:

- wet method synthesis, using the starting materials in the form of salt [55, 57–61] or acids as well as bases and oxides [62];
- the sol-gel technique [63, 64];
- ion exchange between the pure hydroxyapatite and a silver salt solution [59, 65–67];
- ultrasonic spray pyrolysis (USSP) [68];
- the microwave method [69].

The resulting materials may be then subjected to treatment, such as heat-treating by sintering in an oven or drying with the use of the sublimation method. Depending upon the method of synthesis or the possible treatment, the resulting apatites are characterized mainly by their degree of crystallinity and their specific surface area. It affects the efficiency of the antibacterial activity because the rate and extent of the release of silver ions from the apatite increase along with its surface area, and thus techniques that help in obtaining fine crystals will lead to the obtaining of a material exhibiting stronger antibacterial activity. For example, it was proved that drying the silver-hydroxyapatite using the sublimation method leads to the preparation of a material with finer crystals, thereby also enhancing the activity of antibacterial Ag-HAp [57]. In addition, methods of synthesis (such as USSP, the direct incorporation of Ag^+^ ions into the structure of HAp, as well as increases in the crystallinity of Ag-HAp by sintering) lead to the preparation of a material with a slower release of Ag^+^ ions, and thus to prolonged antibacterial activity [68].

During Ag-HAp synthesis, different amounts of silver are introduced within the range of 0.1% to 10% of weight, usually in several increasing concentrations, allowing a series of tests for the various contents of silver. The aim of such a study is to determine the optimum concentrations, characterized by a high efficiency of action against a broad range of microorganisms.

**Figure 6:**
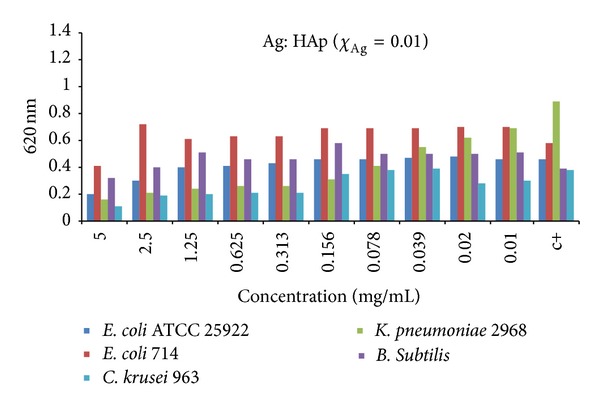
Antibacterial activity of Ag-HAp (*χ*_Ag_ = 0.1) on* E. coli* ATCC 25922,* E. coli* 714,* Klebsiella pneumoniae* 2968,* Bacillus subtilis*, and* Candida krusei* strains (adapted from [69] with permission).

The broad antibacterial spectrum of Ag-HAp is similar and has the same width as the spectrum of silver ions, including bacteria, viruses, and fungi [70]. Moreover, like the silver ions, it has a more effective influence on gram-negative rather than gram-positive bacteria, due to the differences in the structures of cell walls [70]. Even such a low content of silver as 0.2% of weight in Ag-HAp effectively inhibits the growth of* K. pneumoniae* and* C. krusei*. In contrast, antibacterial activity against* E. coli* and* B. subtilis* requires a content of 0.5% weight of silver in the apatite [69] (see Figure 6). The growth of* S. aureus* and* Staphylococcus epidermidis* is clearly inhibited with a silver content of 1% of weight [71].

**Figure 7:**
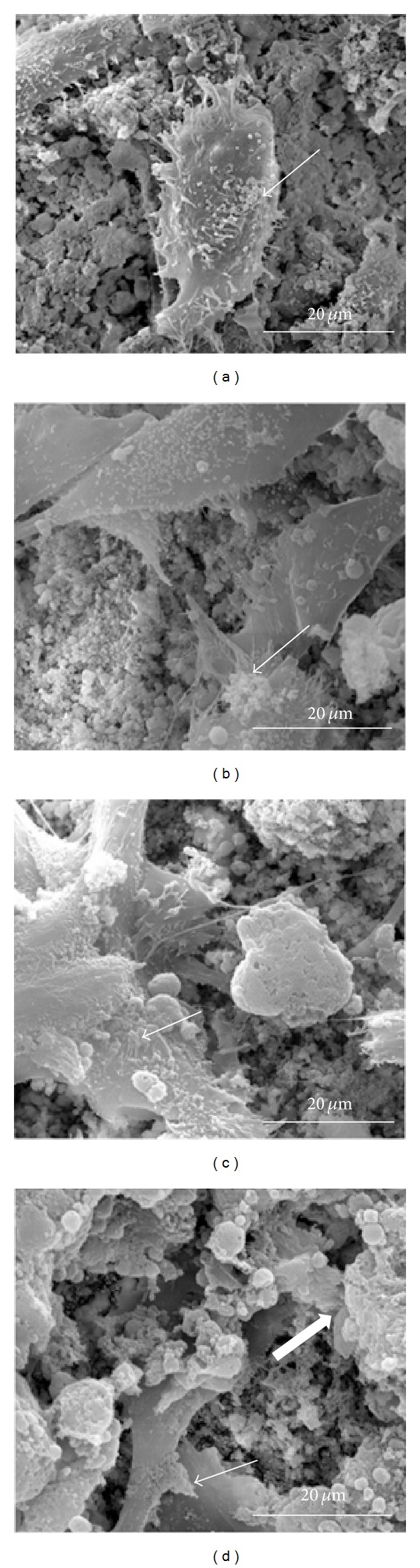
FESEM micrographs illustrating the hFOB cell morphology after three days of culture: (a) HA, (b) HA-2Ag, (c) HA-4Ag, and (d) HA-6Ag. The hairline arrow indicates a live cell and the thick arrow indicates a dead cell (reprinted from [72] with permission).

Excessive silver content in the hydroxyapatite may be toxic, not only to microorganisms but also to mammalian tissues, and may lead to the inhibition of their growth [61, 71, 72]. Therefore, the selection of an appropriate silver content in Ag-HAp is an important issue, since it should be high enough to be able to effectively fight microorganisms, while at the same time it should also be limited in order that it does not adversely affect the condition of mammalian tissues. Numerous toxicological studies have shown that, along with the increase of the content and the rate of release of silver ions from Ag-HAp, we can also observe an increase in their negative impact on tissues, inhibiting their growth and development, and in higher Ag^+^ concentrations—leading to death of the cells. In order to determine the optimum silver content, one performed* in vitro* toxicity studies on human osteoblasts [71, 73, 74], human stem cells [61], and mouse fibroblasts [75], as well as* in vivo* studies in rats involving the implantation of Ag-HAp.* In vitro* studies carried out on human stem cells show that a hydroxyapatite containing 0.3% silver weight has no negative effect on the growth of these cells over the course of seven days of culture, while a content of 0.7% silver slightly inhibits their growth. In contrast, a concentration of 8.3% weight exhibits a significant cytotoxic effect, specifically in inhibiting the growth of human stem cells [61].* In vitro* experiments on human osteoblasts have shown that a silver content of 6% weight significantly inhibits the growth of osteoblasts and leads to the death of some of them, while 2% and 4% weights represent contents that enable a good balance between effective antimicrobial and cytotoxic activity [73]. In addition,* in vitro* studies conducted on rats confirmed the relatively low toxicity of Ag-HAp, containing up to 4.3% weight (see Figure 7) [72].

The complexity of the problem of the balance between effective antimicrobial activity and cytotoxic activity includes not only the total content of silver in the hydroxyapatite but also the conditions of its synthesis and the method for its drying and heat treating. Nevertheless, one can observe a significant and still growing interest in this promising material.

## 3. Cu^2+^-Substituted Hydroxyapatite

Copper is an essential micronutrient of almost all living organisms because it is involved in many metabolic processes. However, higher concentrations of copper ions may have toxic effects,* inter alia*, because of their ability to generate ROSs. The antibacterial properties of copper were discovered very early on; we know of Egyptian texts written around 2600–2200 BC which present methods for the sterilization of drinking water and wounds to the chest using copper [76].

The mechanism of the antibacterial activity of hydroxyapatites doped with copper ions is not yet fully understood. It is believed that copper ions form strong bonds with thiolic, imidazole, amine, and carboxylic groups of proteins, causing structural changes and increases in permeability and, hence, membrane transport dysfunction and cell death [77]. In addition, copper ions also form a bonding with amine and amide groups, as well as with the disulphide bridges of proteins and enzymes of bacteria, causing damage to DNA and RNA and resulting in the inhibition of the reproduction of bacteria or their death [78].

The antibacterial properties of copper are also used by macrophages which, by copper concentration, increase their ability to inactivate microorganisms through the oxygen burst [79]. Therefore, some microorganisms, such as* Mycobacterium tuberculosis*,* S. aureus*, and* Salmonella enterica*, have produced mechanisms protecting them from the toxic effect of copper in order to be able to survive the intervention of macrophages [80–83]. In addition, copper deficiency reduces the bactericidal activity of neutrophils and* in vitro* macrophages [84].

Studies have confirmed the antibacterial efficiency of hydroxyapatites doped with copper ions against* E. coli* bacteria. Hydroxyapatites were synthesized using the wet method. Copper ions were introduced into the structure using copper acetate of Cu (CH_3_COO)_2_·H_2_O. Hydroxyapatites doped with copper ions to the amount 3.3% of weight inhibited the growth of* E. coli* bacteria, while a content of 0.66% of weight of Cu^2+^ was deemed to be insufficient to inhibit bacteria [85].

**Figure 8:**
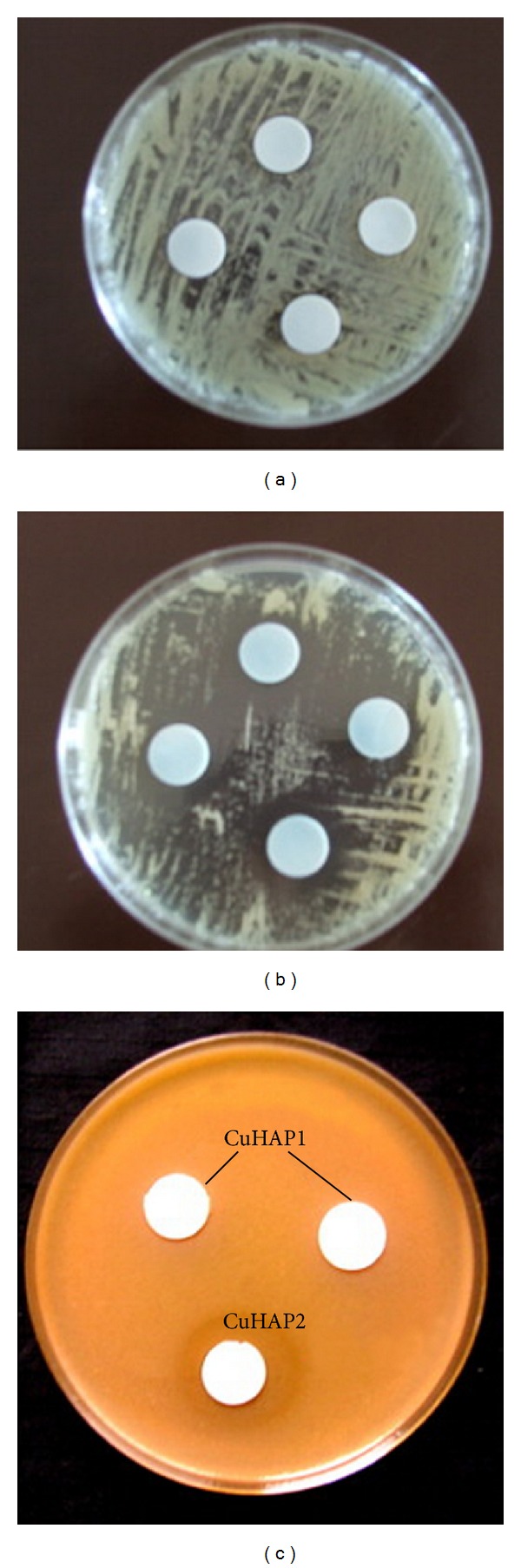
Photographs of the antimicrobial test results of CuHAP samples against* E. coli*: (a) CuHAP1, (b) CuHAP2, and* C. albicans*: (c) CuHAP1 and CuHAP2 (reprinted from [77] with permission).

Studies have also shown the inhibitory effect of hydroxyapatites doped with copper ions on the growth of* Candida albicans* fungi [77] (see Figure 8).

In this case, hydroxyapatites were also obtained using the wet method, while the copper ion source was copper oxide (II) CuO. The resulting material more strongly inhibited the growth of fungi than in the case of studies of hydroxyapatites doped with zinc ions. Copper ions do not inhibit the multiplication of* S. aureus* bacteria, which is probably due to the fact that* S. aureus* is a gram-positive bacteria.

Cooper ions, in contrast to other divalent metal cations (Zn, Co, Ni, Mn, Fe, and Sn), cause selective changes in the permeability of the cell membrane of the* Saccharomyces cerevisiae* yeasts, with no changes in the vacuole membrane permeability [86].

In the case of hydroxyapatites doped with copper ions, a problem may be caused by the cytotoxic properties of copper, both against bacteria and osteoblasts [77]. Such an effect can be observed in the case of hydroxyapatites with a content of 0.66% of weight of Cu^2+^.

## 4. Zn^2+^-Substituted Hydroxyapatite

Zinc, one of the essential microelements, is present in the active centres of more than 300 enzymes involved in the metabolism of bones. It also exhibits antibacterial activity, the mechanism of which is similar to that of copper ions. Due to its antibacterial properties, one started to introduce zinc ions into the molecule of hydroxyapatite. Moreover, zinc ions have also had a direct impact on the proliferative properties of osteoblasts and an inhibitory effect on bone resorption by osteoclasts.

Some papers have presented studies on hydroxyapatites doped with small amounts of zinc ions (less than 1%), which confirmed effective bioactivity and antibacterial properties [77, 85–90]. In contrast, 1.3% content of zinc ions causes an increase in osteoblast responses [91]. In addition to their biological properties, one has also confirmed the inhibitory effect of hydroxyapatites doped with zinc on the development of bacteria and fungi, including* E. coli*,* S. aureus*,* C. albicans*, and* Streptococcus mutans* [92]. Studies on a hydroxyapatite doped with zinc, synthesized using the wet method and a hexahydrate zinc nitrate of Zn (NO_3_)_2_·6H_2_O (wherein the zinc ion content was 1.6%), have shown its real impact on the reduction of the numbers of* S. aureus* bacteria [93]. In addition, it was proved that the small amount of zinc ions introduced into the hydroxyapatite structure plays an important role in the processes of the growth and differentiation of the cells; however, the mechanism of the ions' impact on biological processes has not yet been discovered.

The use of hydroxyapatites doped with zinc ions as a coating for the remineralization of teeth helps to reduce bacterial adherence and the growth of tartar. Studies confirmed the efficiency of hydroxyapatites doped with zinc ions in reducing the growth of the three most common oral pathogens, including* Aggregatibacter actinomycetemcomitans*,* Fusobacterium nucleatum*, and* S. mutans* [94] (see Table 1).

Hydroxyapatites are also used in the treatment of bone cancer and chronic osteomyelitis. In these and other diseases of the bones, infections pose serious problems. Unfortunately, the most commonly used antibiotics have very low concentrations in the bone tissue. Therefore, it is crucial to look for a solution which achieves high concentrations of antibiotics in the bone tissue while reducing the toxic effect of drugs on the human body. Studies have been performed on the efficiency of hydroxyapatites doped with zinc ions as carriers for the controlled release of ciprofloxacin, which exhibits antibacterial activity against* S. aureus *and* Pseudomonas aeruginosa* (these being the most common pathogens causing diseases of the bones and joints). They have shown that the release of ciprofloxacin by the hydroxyapatite takes place in connection with the release of zinc ions and increases in the drug concentration and number of Zn^2+^ ions used increases the antibacterial activity [95]. Furthermore, the release of ciprofloxacin from hydroxyapatite doped with zinc ions is greater than the drug released from the “pure” hydroxyapatite. The use of such a solution in the treatment of infections of bone tissue allows for the shortening of therapy—increasing its efficiency—and the reduction of the dose of ciprofloxacin, which contributes to the reduction of the formation of resistant strains. As in the case of hydroxyapatites doped with copper ions, the use of hydroxyapatites containing zinc ions may be limited by the cytotoxicity of zinc, which is revealed when the content exceeds 1.2% [93].

Due to the possibly identical mechanism of the antibacterial activity of zinc and copper ions and their similar efficiency as regards the treatment of microorganisms, the studies on the simultaneous introduction of the ions of these both metals into the hydroxyapatite structure are conducted.

## 5. SeO_3_^2−^-Substituted Hydroxyapatite

Selenium is one of the microelements necessary for the proper development of the human body. Yet, until recently, it was considered a highly toxic agent [96, 97]. However, studies have shown that it is a component of selenoproteins and the glutathione peroxidase enzyme, responsible for the protection of cell membranes against harmful factors [97]. Many studies have proved that selenium is a factor which protects against oxidative stress and carcinogenesis. Therefore, one performs research on the use of selenium compounds in the production of multifunctional biomaterials which, in addition to the scaffold function, also represent the anticarcinogenic functions [98]. Studies conducted by Tran et al. [99] and Rodríguez-Valencia et al. [100] have also shown favourable antibacterial properties. Selenium ions of SeO_3_^2−^ were introduced into the carbonated hydroxyapatite structure using the pulsed laser deposition method (PLD). The material formed this way was used to create a coating for titanium. Its antibacterial activity on the strains of* S. aureus* and* P. aeruginosa *was found [100]. Even at a concentration of 0.6% weight of selenium in the coating, there was an inhibition of biofilm formation by the two bacterial strains. It is worth noting that the concentration of selenium was nontoxic and had no adverse effect on the adhesion of the cells or their proliferation. The literature discusses the antibacterial mechanism of selenium [100–103]. It is most likely associated with oxidative stress, resulting in damage to the cell walls of bacteria. Microorganisms metabolize selenium to a harmless form of elemental selenium of Se^0^ or HSe^−^ (hydrogen selenide), which in turn can be included in selenocysteine. As a result of metabolic reactions—which are oxidation-reduction reactions—H_2_O_2_ or even O_2_^−^ may be created, which would confirm the theory of the oxidative antibacterial mechanism of selenium.

## 6. Sr^2+^-Substituted Hydroxyapatite

Strontium is a microelement that cumulates in the bone mineral, playing an important role in it. A small amount of strontium induces the formation of bone tissue and the inhibition of its resorption. The available literature also contains information about the relief of bone pains and even attempts to treat bone cancer [104, 105]. There are many elaborations devoted to the introduction of strontium ions into the hydroxyapatite structure [106–109]. Due to their similar properties and small differences in size, calcium cations may be completely replaced with strontium cations. The introduction of strontium ions into the hydroxyapatite improves its biocompatibility and bioactivity, streamlining the process of osseointegration. In addition, strontium ions improve the mechanical properties of the hydroxyapatite.

Recent reports also show that strontium can be used as an antibacterial agent. Guida et al. [110] suggested that the antibacterial activity of glass-ionomer cements is mainly caused by the presence of strontium and not fluoride, as was previously thought. In turn, in the paper of [111], it was proved that the antibacterial properties of strontium are very poor but that its combination with fluoride improves its antibacterial potential in the treatment of tooth decay.

One also studied the antibacterial activity of hydroxyapatite incorporated with strontium ions. In the elaboration of [112], where Sr-hydroxyapatite was synthesized using the sol-gel-supercritical fluid drying method (SCFD), it was proved that the material containing strontium ions exhibited antibacterial activity against* E. coli*,* S. aureus*, and* Lactobacillus*. In turn, in the elaboration of [113], Sr-HAp was prepared using the accelerating action of microwaves. A small amount of strontium in the received materials caused a bactericidal effect on* E. coli* and* S. aureus*.

## 7. Ce^3+^- and Eu^3+^-Doped Hydroxyapatite

Cerium and europium are rare earth elements from lanthanides, which are present in the human body only as a result of the cumulative effect of the environment. They have the ability to accumulate in small amounts in the bones and liver. We do not know the biological role of cerium or europium, but recent studies have shown that cerium salts may stimulate metabolism [114].

In recent years, researchers have begun to investigate the biological properties of the cerium oxide of CeO_2_. It has been proven that cerium oxide is able to induce angiogenesis through its direct effect on the modulation of oxygen in intracellular environments [115]. In turn, Lord et al. [116] have studied the impact of cerium oxide on human monocytes, based on its ability to scavenge ROSs. The antibacterial properties of cerium oxide have also been studied in a number of elaborations, especially of strains of* E. coli*,* B. subtilis*,* Salmonella typhimurium*, and* Enterococcus faecalis* [117, 118].

There are few works devoted to the antibacterial properties of cerium ions and their use in biomaterials engineering. However, the literature contains reports on the synergistic effect of the activity of zinc and cerium ions in nanomaterials of alpha-zirconium phosphate and titanium [119, 120].

In the available literature, only two elaborations are devoted to hydroxyapatite doped with cerium ions [121, 122]. Feng et al. synthesized hydroxyapatite containing a maximum of 10% weight of cerium using the hydrothermal method [121]. The introduction of cerium ions to the hydroxyapatite structure was manifested by the reduction in the crystal size to about 20 nm. Unfortunately, this elaboration did not consider research into the antibacterial properties of the resulting material. Lin et al. [122] synthesized hydroxyapatite containing cerium ions using the sol-gel-supercritical fluid drying method (SCFD). The introduction of cerium ions into the structure resulted in the reduction of the crystallinity of the materials and a change in crystal morphology: from rod-shaped HAp to needle-shaped Ce-HAp. The elaboration was devoted to the antibacterial activity of the resulting materials based on the following bacterial strains:* E. coli*,* S. aureus*, and* Lactobacillus*. It turned out that the materials were characterized by a high antibacterial capacity—the greater the degree of the substitution of calcium ions by cerium ions, the higher the antibacterial capacity. At a low content of cerium in a sample, the antibacterial activity was based only on the dynamic contact of the material with bacteria at a concentration of 0.1 g/mL.

**Figure 9:**
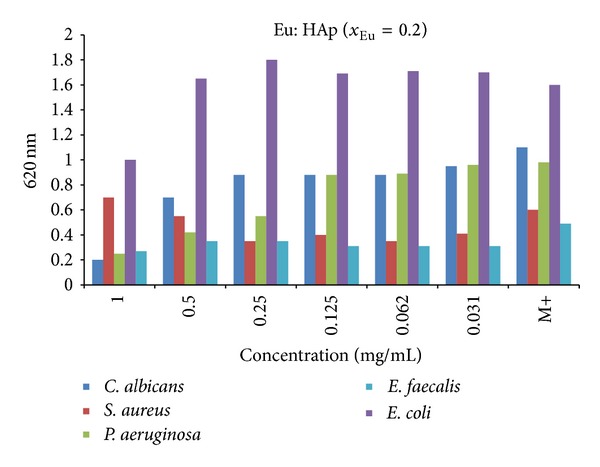
Antimicrobial activity of as-prepared Eu:HAp samples (*χ*_Eu_ = 0.2) on* E. coli* ATCC 25922,* P. aeruginosa* 1397,* S. aureus* 0364,* E. faecalis* ATCC 29212, and* C. albicans* ATCC 10231 (adapted from [123] with permission).

When it comes to the antibacterial activity of europium ions in the context of biomaterials, it has been described in [123, 124]. One studied the antibacterial activity of materials synthesized using the coprecipitation method (i.e., the wet method) on the following strains of bacteria and fungi:* S. aureus*,* E. coli*,* P. aeruginosa*,* E. faecalis*, and* C. albicans* [123] (see Figure 9). These studies have shown that even with a low content of Eu^3+^, the materials had an antibacterial effect on strains of* P. aeruginosa*,* S. aureus,* and* E. faecalis*. In contrast, these materials showed no bactericidal capacity in relation to the* E. coli* strain. Fungicidal activity was exhibited by the materials with a higher content of europium.

## 8. Ga^3+^-Substituted Hydroxyapatite

Gallium is an element from the group of semimetals. Its efficacy in the treatment of many types of disorders has been developed in the late 20th century. A wide range of potential applications have been described in an extensive review of Bernstein [125]. Gallium inhibits bone resorption and thus lowers the calcium concentration in plasma. In addition, it induces osteoblasts and helps bone formation. It has a positive effect on the adoption of implants. Numerous studies have also proved its immunomodulatory activity. In addition, gallium—due to its antiproliferative and antimitotic effect—may positively influence the treatment of certain types of cancer [125].

Due to the high similarity of Fe^3+^ ions (i.e., similar ionic radius, electronegativity, coordination number, etc.), gallium ions of Ga^3+^ may replace them in a variety of metabolic reactions. With this property, gallium is used as a diagnostic and therapeutic agent in metabolic disorders of soft and hard tissues [125].

Recent studies have also proved the antibacterial activity of gallium ions, which is based on the exchange of iron ions in protein metabolism [126, 127]. One showed the antibacterial activity of phosphorus, silica, and phosphorus glass containing gallium.

The available literature contains information on hydroxyapatite synthesis, enriched with gallium ions III [128]. The content in the material amounted to 11% of weight. However, one has not examined the antibacterial activity of this material. In our opinion, such studies should be carried out.

## 9. Ti^4+^-Containing Hydroxyapatite

In recent years, a lot of attention has been given to titanium oxide TiO_2_, due to its photocatalytic properties [129, 130]. One uses its photooxidative activity on organic materials, such as proteins and lipids. The mechanism of photocatalysis of titanium oxide is described in the elaboration of [131]. It takes place according to the following reactions:

(1) $$\begin{array}{c} {\text{TiO}}_{2}+\text{h}\nu ⟶{{\text{e}}^{-}}_{\text{CB}}+{{\text{h}}^{+}}_{\text{VB}} \end{array}$$

**Figure 10:**
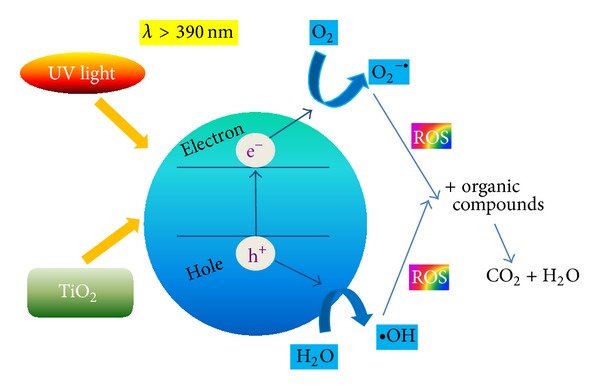
Photocatalytic properties of TiO_2_ and its antibacterial potential (adapted from [132].

Free electrons (e^−^_CB_) and positively charged holes (h^+^_VB_) have strong oxidizing and reducing properties. When electrons and vacancies migrate to the surface, TiO_2_ can participate in the reactions of oxidation/reduction with molecules absorbed on the surface (i.e., water, oxygen, and organic and inorganic molecules) (Figure 10). Electrons may reduce Ti(IV) to Ti(III), which in turn reacts with the oxygen to form O_2_^−•^ radicals. Vacancies may react with water or ions of OH^−^ and form radicals of •OH:

(2) $$\begin{array}{c} {\text{H}}_{2}\text{O}+{{\text{h}}^{+}}_{\text{VB}}⟶•\text{OH}+{\text{H}}^{+}\\ {\text{OH}}^{-}+{{\text{h}}^{+}}_{\text{VB}}⟶•\text{OH} \end{array}$$

In turn, the radicals of O_2_^−•^ and •OH may react together, causing the formation of H_2_O_2_.

These factors, the so-called “reactive oxygen species” (ROSs), may react with the wall or membrane of the cell of bacteria. Unsaturated phospholipids—contained in these cell elements—participate in reactions with ROS, causing the death of the bacterial cell.

The antibacterial properties of Ti(IV) are used in biomaterials engineering. In addition to the use of titanium oxide as a component of biomaterial components, the literature describes hydroxyapatites doped with Ti^4+^ ions [133, 134]. Wakamura [133] proved that Ti^4+^ in hydroxyapatite—where there are small amounts of Ti (molar ratio of Ca/P to 100)—forms divalent cations [Ti(OH)_2_]^2+^ and [TiHPO_4_]^2+^, which replace calcium cations. Titanium contained in hydroxyapatite crystals has a similar antibacterial mechanism to titanium oxide. Hydroxyapatite has a high affinity for proteins; therefore, hydroxyapatite modified with Ti^4+^ ions has an antibacterial effect not only after UV irradiation.

## 10. Co^2+^-Substituted Hydroxyapatite

Cobalt is an essential element for the proper functioning of the human body. First of all, it is a component of vitamin B12, which is necessary for the regulation of the production of red blood cells, DNA synthesis in cells, and the formation of the myelin sheath, protecting the cells of nerves and neurotransmitters [135].

The literature presents information on the use of Co (II) ions as an antibacterial and antiviral agent in various organic complexes [136, 137]. In 2013, two elaborations were developed concerning hydroxyapatites partly doped with cobalt ions (II) [138, 139]. In their work, Ignjatović et al. [138] presented the hydrothermal method for obtaining hydroxyapatite containing Co^2+^ at the amount of 5–15% of weight. Unfortunately, so far, nobody has conducted studies on the antibacterial activity of the resulting materials. In turn, Stânila et al. [139] have synthesized nanocrystalline hydroxyapatite enriched with Co^2+^ ions using the wet method and obtained the materials containing between 0.46% and 3.79% of cobalt weight. The resultant cobalt ion concentrations were lower than expected before the synthesis, which was most likely related to differences in the constant bonding and chemical affinity of the ions of cobalt (II) and calcium (II). The antibacterial activity was tested on the basis of strains of the following bacteria:* S. aureus*,* Micrococcus luteus*,* P. aeruginosa*, and* Shigella flexneri*. Nanocrystalline hydroxyapatite was used as a comparative material, obtained under the same conditions. In studies carried out in the elaboration of [139], it turned out that all the tested materials (even nanocrystalline HAp) exhibited antibacterial activity against the studied strains, with the exception of* P. aeruginosa* and with the proviso that this activity strongly increased along with an increase of the cobalt content of the material. It is also worth noting that the materials were not toxic (according to haemolysis assay), which gives hope for their use in medicine as biomaterials with additional antibacterial properties.

## 11. Conclusions

Hydroxyapatite plays a key role in bioceramic and biomaterial composites and is commonly used in reconstruction medicine and dental implantology. However, we can observe a trend of a broadening of the spectrum of the apatite materials, with additional biological, physicochemical, or biomechanical properties, despite the scaffold for the newly formed bone. It seems very reasonable to look for the possibility of the substitution of ions with antibacterial properties. So far, the best-known and most commonly used material is hydroxyapatite enriched with silver ions. Much is also known about the antibacterial activity of hydroxyapatites substituted by the ions of copper and zinc. On the other hand, it seems that the other materials still require a great deal of research. In our opinion, one should also examine the relationship between the method of the preparation of the substituted apatite material with its antibacterial activity and its efficiency. Such studies remain very rare or else incomplete.

## Figures and Tables

**Table 1:** Antibacterial activities of zinc-loaded calcium phosphate nanostructures. The samples A, B, and C were prepared by different routes: A—the obtained precipitate was dried and heated at 200°C for 3 h before Zn-loading; B—the collected wet product was directly used for Zn-loading; and C—prepared at 70°C for 10 h (rewritten from [94] with permission).

Sample	Bacterium	Inhibition rate %	Bacterium	Inhibition rate %	Bacterium	Inhibition rate %
A	*Aggregatibacter actinomycetemcomitans *	99.82	*Fusobacterium nucleatum *	8.91	*Streptococcus mutans *	17.25
B		95.98		99.97		86.87
C		>99.99		>99.99		>99.99
